# Live Birth Outcomes After Extended or Repeated High‐Dose Medroxyprogesterone Acetate Therapy for Fertility‐Sparing Management of Endometrial Neoplasia: A Single‐Center Retrospective Case Series

**DOI:** 10.1111/jog.70178

**Published:** 2026-01-14

**Authors:** Akitoshi Yamamura, Asuka Okunomiya, Akihiro Yanai, Koji Yamanoi, Mana Taki, Tsutomu Ohara, Taito Miyamoto, Masumi Sunada, Yukiko Okada, Masaki Mandai

**Affiliations:** ^1^ Department of Gynecology and Obstetrics Kyoto University Graduate School of Medicine Kyoto Japan; ^2^ Department of Obstetrics and Gynecology Shizuoka General Hospital Shizuoka Japan

**Keywords:** atypical endometrial hyperplasia, endometrial cancer, fertility, live birth, medroxyprogesterone acetate

## Abstract

**Aim:**

To clarify live birth outcomes among women receiving extended or repeated high‐dose medroxyprogesterone acetate (MPA) therapy for fertility‐sparing management of atypical endometrial hyperplasia or endometrioid carcinoma grade 1.

**Methods:**

We conducted a single‐center retrospective case series of 53 patients undergoing MPA therapy between 2005 and 2023. Patients were stratified into three groups: (i) complete response (CR) within 6 months (standard group), (ii) CR after extended treatment beyond 6 months (extended group), and (iii) CR after MPA retreatment for first intrauterine recurrence (retreatment group). Primary outcome was the live birth rate (LBR). Secondary outcomes included the effect of initial reproductive intentions, interval from CR to conception, recurrence rates, and recurrence‐free interval (RFI).

**Results:**

LBRs were 33% (10/30) in the standard group, 8% (1/12) in the extended group, and 17% (2/12) in the retreatment group. Among eight patients undergoing MPA retreatment for a second or subsequent recurrence, none achieved live birth. Patients with an initial desire for prompt conception had significantly higher LBRs than those without (38% vs. 5%, *p* < 0.01). Median time from CR to conception leading to live birth was 12 months. Patients achieving live birth had significantly longer RFIs than those without (*p* < 0.01).

**Conclusions:**

Live birth is most likely when CR is achieved within 6 months of MPA therapy; nonetheless, extended or repeated MPA treatment may still result in live birth. These findings suggest the importance of appropriate patient selection and careful monitoring during extended or repeated therapy and attempting conception promptly in fertility‐sparing management of endometrial neoplasia.

## 1 Introduction

Endometrial cancer is the most common gynecological malignancy in developed countries, and its incidence is increasing [1]. Approximately 9.5% of endometrial cancers occur in women younger than 40 years [2], many of whom still desire fertility‐sparing treatment. The standard treatment for endometrial cancer is total hysterectomy. However, with the increasing age at first childbirth in developed countries, the need for fertility‐sparing treatment is growing.

High‐dose medroxyprogesterone acetate (MPA) therapy is a fertility‐sparing treatment option for endometrioid carcinoma grade 1 confined to the endometrium and its precursor lesion, atypical endometrial hyperplasia (AEH). Because MPA therapy is used to treat malignant or premalignant lesions, a sufficient anti‐tumor effect is crucial. Although a response rate of 70%–80% has been achieved, a relatively high recurrence rate of approximately 35% has been reported [3].

In MPA therapy, 400–600 mg MPA is typically administered daily for 6 months. Primarily from an anti‐tumor perspective, the guidelines in Japan, as well as those from the European Society of Gynecological Oncology (ESGO), European Society for Radiotherapy and Oncology (ESTRO), and European Society of Pathology (ESP), recommend total hysterectomy if a complete response (CR) is not achieved after 6 months of treatment [4, 5]. The National Comprehensive Cancer Network (NCCN) guidelines recommend total hysterectomy if the lesion persists after 6–12 months of treatment and recommend reevaluation and repeated imaging when continuing MPA therapy beyond 6 months [6]. Regarding MPA retreatment for intrauterine recurrence after MPA therapy, several series have reported high second CR rates of approximately 80%–100% [7, 8, 9]. In light of these findings, the Japanese guidelines state that although MPA retreatment for recurrence is not considered standard therapy, it may be considered under careful clinical surveillance [4]. In contrast, the NCCN guidelines do not recommend this strategy [6]. Regardless, in real‐world practice, a proportion of patients strongly desire retreatment after recurrence. Therefore, evaluating the oncological and reproductive outcomes of MPA retreatment in such patients remains clinically meaningful.

This study aimed to clarify live birth outcomes among patients receiving extended or repeated MPA therapy, compared with those achieving CR within 6 months. We further examined recurrence, recurrence‐free interval (RFI), and the effect of initial reproductive intentions to provide practical information for patient counseling.

## 2 Methods

### 2.1 Study Design

This retrospective case series was conducted at Kyoto University Hospital. Records from January 2005 to December 2023 were reviewed. The study was approved by the institutional ethics committee (approval no. R4535). Information regarding the study was disclosed on the hospital's website to allow patients the opportunity to opt out. The study complied with the Declaration of Helsinki and relevant Japanese laws and ethical guidelines.

### 2.2 Study Populations

We included patients diagnosed with AEH or endometrioid carcinoma grade 1 confined to the endometrium at Kyoto University Hospital between January 1, 2005, and December 31, 2023, who underwent high‐dose MPA therapy as a fertility‐sparing treatment. The exclusion criteria were as follows: (1) cases in which the outcome of the initial treatment had not been evaluated on December 31, 2023, and (2) cases that deviated from our institution's standard treatment protocol, as described below.

### 2.3 Treatment Protocols

Patients pathologically diagnosed with AEH or endometrioid carcinoma grade 1 after hysteroscopy and endometrial curettage were further evaluated with pelvic magnetic resonance imaging to confirm the absence of myometrial invasion or intrapelvic spread. For endometrioid carcinoma cases, computed tomography was additionally performed to exclude distant metastasis or lymph node involvement. After confirmation on these radiological assessments, the patients received 600 mg/day MPA and 100 mg/day aspirin for 6 months. Hysteroscopy and endometrial curettage were performed at 3 and 6 months to examine the therapeutic effects. If CR was not achieved at 6 months of initial treatment, total hysterectomy was recommended in principle. If the patient strongly desired a fertility‐sparing approach and some pathological response to MPA was observed, MPA therapy was extended with hysteroscopic re‐evaluations every 3 months. The pathological response to MPA was determined on a case‐by‐case basis through discussion between gynecologists and gynecologic pathologists, using an integrated assessment of reduced lesion extent, improvement in glandular architecture, amelioration of cytologic atypia, and stromal decidualization.

Recurrence was defined as the reappearance of AEH or a more advanced lesion after achieving CR. In cases of recurrence after achieving CR, total hysterectomy was recommended in principle. If patients strongly desired fertility‐sparing management, MPA retreatment was considered using the same criteria and assessment schedule as the initial treatment only when they were able to adhere to the surveillance schedule we recommended.

### 2.4 Outcome Measures and Data Collection

#### 2.4.1 Primary Outcome

Live birth rate (LBR). We examined the following three groups:

(i) Those who achieved CR within 6 months (standard group), (ii) those who achieved CR beyond 6 months (extended group), and (iii) those who achieved a second CR through MPA retreatment for recurrence (retreatment group).

#### 2.4.2 Secondary Outcomes

The relationship between the desire for prompt conception at the first visit and the LBR, interval from CR to conception (confirmation of a gestational sac), recurrence rate, and RFI.

### 2.5 Statistical Analysis

Statistical analyses were performed using R (version 4.1.1) and EZR [10]. Continuous variables were compared using the Mann–Whitney U test and are presented as medians with ranges. Categorical variables were compared using Fisher's exact test. The 95% confidence intervals (CIs) for LBRs were calculated using the Wilson score method. Odds ratios (ORs) with 95% CIs were estimated from 2 × 2 contingency tables. The RFI was analyzed using the Kaplan–Meier method, and group differences were assessed using the log‐rank test. A significance level of 5% was used, and all *p* values were two‐sided. The Bonferroni correction was applied for multiple comparisons. All analyses were performed using de‐identified individual patient data, which are provided in File S1.

## 3 Results

**FIGURE 1:**
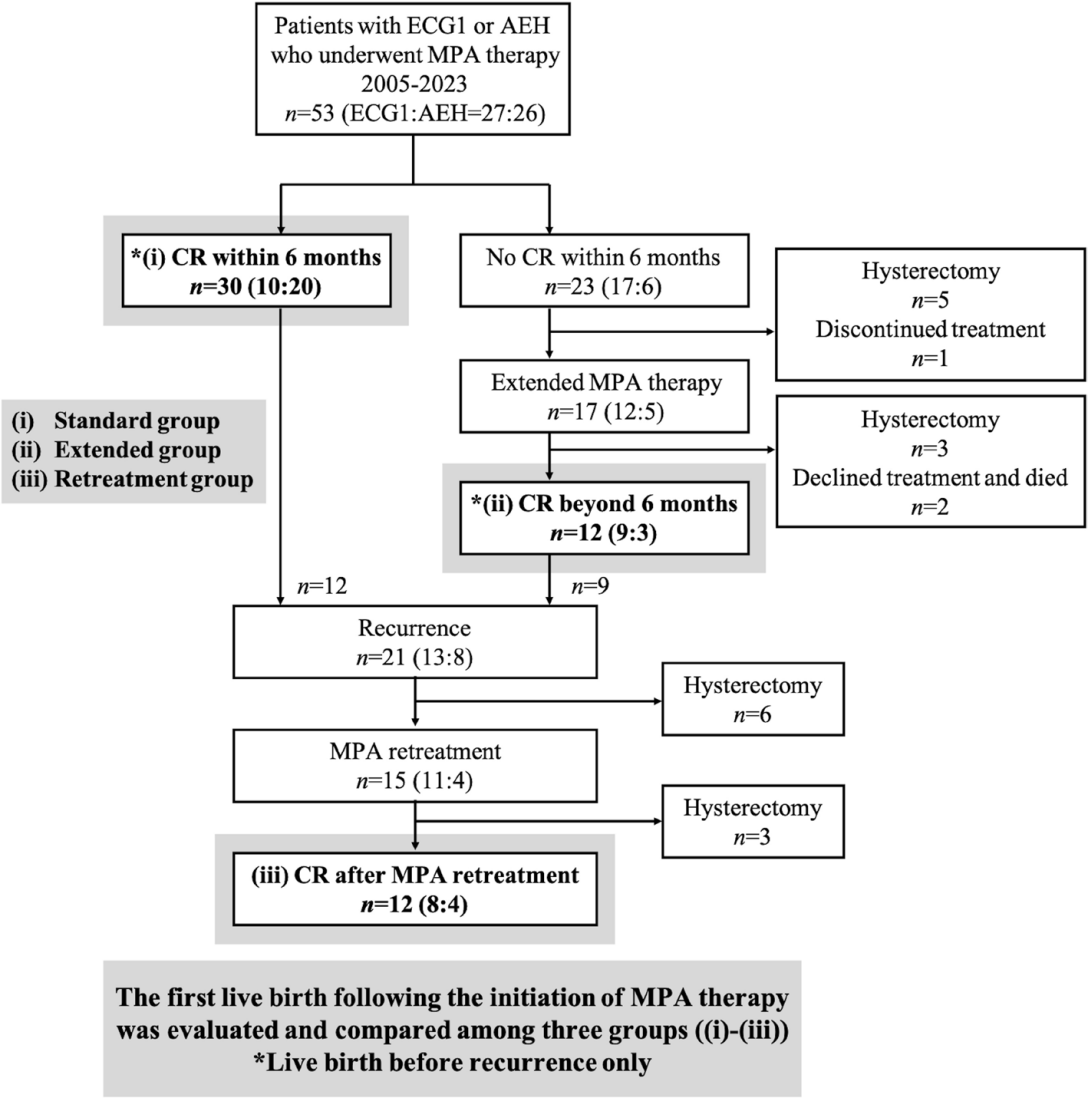
Study flow diagram. Patients were analyzed according to the flowchart. Live birth rates were evaluated based on the duration of MPA therapy required to achieve CR and in patients who experienced recurrence. Along with the total number of cases, the numbers in parentheses indicate ECG1 and AEH case counts, respectively (diagnoses at the initial presentation). AEH, atypical endometrial hyperplasia; CR, complete response; ECG1, endometrial cancer grade 1; MPA, medroxyprogesterone acetate.

The clinical course of 53 patients after the initiation of MPA therapy is shown in Figure 1. Thirty patients (57%) achieved CR within 6 months (i, standard group). Among those who did not achieve CR within 6 months, 17 continued MPA therapy, and 12 subsequently achieved CR beyond 6 months (ii, extended group). Recurrence was observed in 21 patients (50%), of whom 15 were retreated with MPA and 12 achieved second CR (iii, retreatment group). In patients receiving MPA therapy for AEH, one case was upgraded to endometrioid carcinoma grade 2 at 6 months after treatment initiation. No other cases showed progression to lesions beyond endometrioid carcinoma grade 1 during treatment, and no extrauterine spread or lymph node metastasis was observed. All patients who underwent hysterectomy at the physician‐recommended time, owing to a lack of response, showed no evidence of disease post‐hysterectomy. In contrast, two patients who declined hysterectomy despite a poor response eventually died.

The patient characteristics and clinical courses are presented in Table 1. Median age was 34 years. The standard group had a higher proportion of AEH than the extended or retreatment groups.

**TABLE 1:** Patient characteristics and treatment outcomes by group.

	Overall (*n* = 53)	(i) Standard group (*n* = 30)	(ii) Extended group (*n* = 12)	(iii) Retreatment group (*n* = 12)	Adj. *p* (i) vs. (ii)	Adj. *p* (i) vs. (iii)
Age, years	34 [26–44]	34 [27–44]	33 [27–42]	29 [27–35]	1.000	< 0.05
BMI, kg/m^2^	22.2 [16.8–39.0]	22.6 [19.2–39.0]	22.2 [16.8–36.7]	22.2 [19.3–35.2]	1.000	1.000
Married	33 (62)	21 (70)	7 (58)	6 (50)	0.982	0.584
Nulliparity	53 (100)	30 (100)	12 (100)	12 (100)	1.000	1.000
Irregular menstruation before MPA	29 (55)	15 (50)	6 (50)	5 (42)	1.000	1.000
PCO	11 (21)	4 (13)	3 (25)	2 (17)	0.775	1.000
Endometriosis	9 (17)	3 (10)	2 (17)	1 (8)	1.000	1.000
Initial desire for prompt conception	32 (60)	20 (67)	7 (58)	5 (42)	1.000	0.349
Histology of tumor						
AEH	26 (49)	20 (67)	3 (25)	4 (33)	< 0.05	0.167
ECG1	27 (51)	10 (33)	9 (75)	8 (67)		
Infertility treatment before diagnosis	21 (40)	12 (40)	4 (33)	2 (17)	1.000	0.553
Infertility treatment after MPA						
None	20 (38)	9 (30)	5 (42)	6 (50)	1.000	0.800
Non‐ART	12 (23)	7 (23)	3 (25)	1 (8)		
ART	21 (40)	14 (47)	4 (33)	5 (42)		
Live birth	13 (25)	10 (33)	1 (8)	2 (17)	0.134	0.554
ECG1:AEH at initial presentation	4:9	1:9	1:0	2:0		
ART conception	10	9	0	1		
Interval from MPA completion to conception leading to live birth^a^, months	12 [2–58]	13 [3–58]	12 [12–12]	23 [2–43]	1.000	1.000

*Note:* Categorical variables are presented as *n* (%), and continuous variables as medians [range]. *p* values were adjusted using the Bonferroni method. Standard group: patients who achieved CR within 6 months; Extended group: patients who achieved CR beyond 6 months; Retreatment group: patients who achieved a second CR after MPA retreatment for recurrence.

Abbreviations: Adj. *p*, adjusted *p* value; AEH, atypical endometrial hyperplasia; ART, assisted reproductive technology; BMI, body mass index; CR, complete response; ECG1, endometrioid carcinoma grade 1; MPA, medroxyprogesterone acetate; PCO, polycystic ovaries.

^a^ Interval from final MPA administration to gestational week 5.

Overall, 13 patients (25%) achieved live births. Among the 30 patients in the standard group, 10 (33%, 95% CI: 19%–51%) achieved live births after the initial treatment. In contrast, only 1 of the 12 patients in the extended group (8%, 95% CI: 1%–35%) reported a live birth, suggesting a trend toward a lower LBR (OR: 0.19, 95% CI: 0.0039–1.7). Furthermore, 2 of the 12 patients in the retreatment group (17%, 95% CI: 5%–45%) achieved live births [Table 1]. Among 8 patients retreated for a second or subsequent recurrence, none achieved a live birth.

**FIGURE 2:**
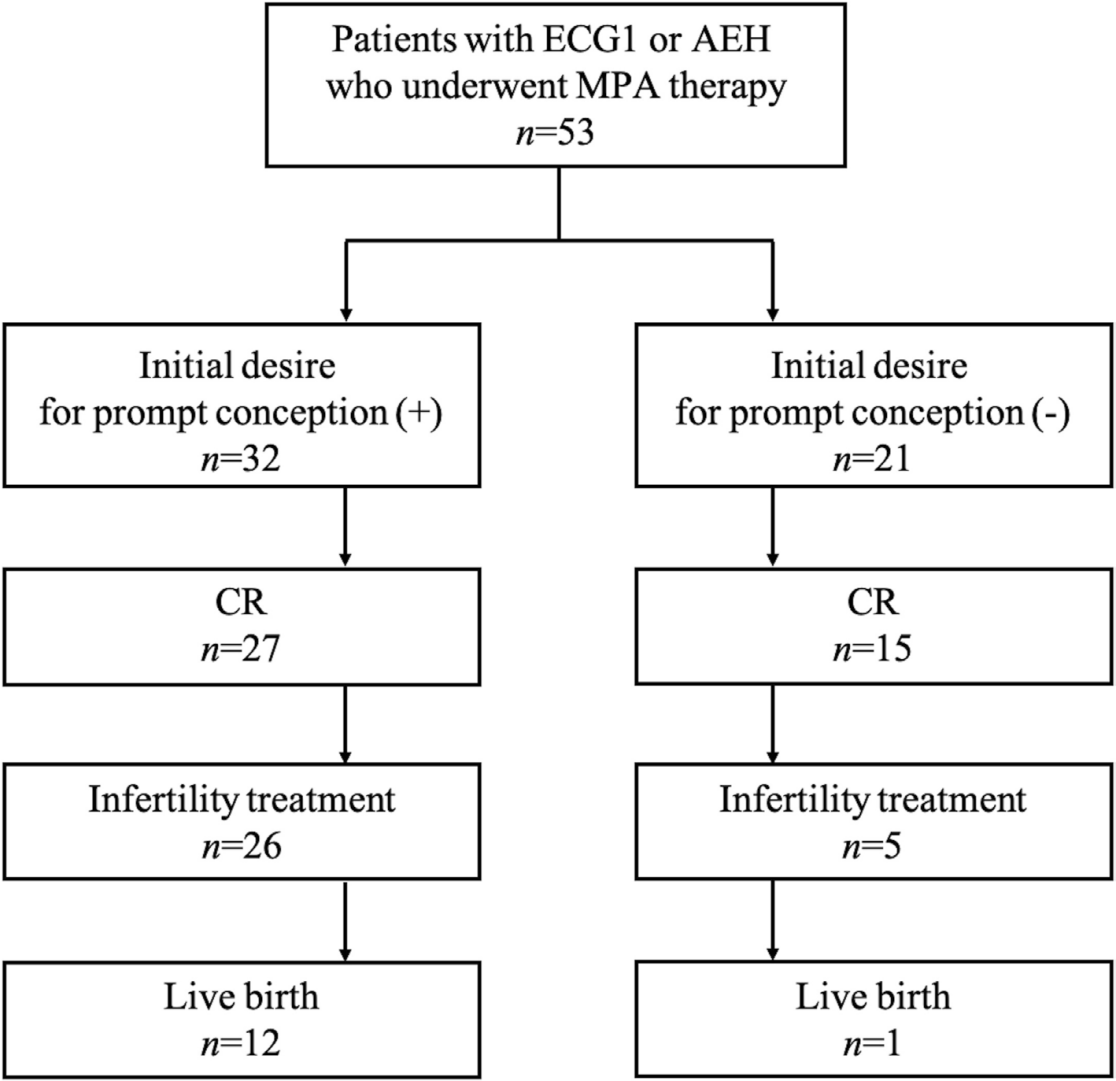
Comparison of final live birth rate according to initial desire for prompt conception. The number of patients who achieved CR after the initial treatment (regardless of treatment duration), the number who subsequently underwent infertility treatment, and the number who ultimately achieved a live birth were counted in each group. Patients who expressed an initial desire for prompt conception show significantly higher live birth rates than those who did not. AEH, atypical endometrial hyperplasia; CR, complete response; ECG1, endometrial cancer grade 1; MPA, medroxyprogesterone acetate.

Among the 32 patients who expressed a desire for prompt conception at their initial visit, 12 (38%, 95% CI: 23%–55%) had a live birth after achieving CR. In contrast, only one of the 21 patients (5%, 95% CI: 1%–23%) who wished to receive fertility‐sparing treatment but did not express a desire for prompt conception, many of whom did not have a partner, achieved live birth. This difference was statistically significant (OR: 11.5, 95% CI: 1.45–537, *p* < 0.01) (Figure 2). The details of infertility treatments performed in the two groups are summarized in Table S1.

The median time from CR to pregnancy resulting in live births was 12 months (2–58 months) (Table 1).

**FIGURE 3:**
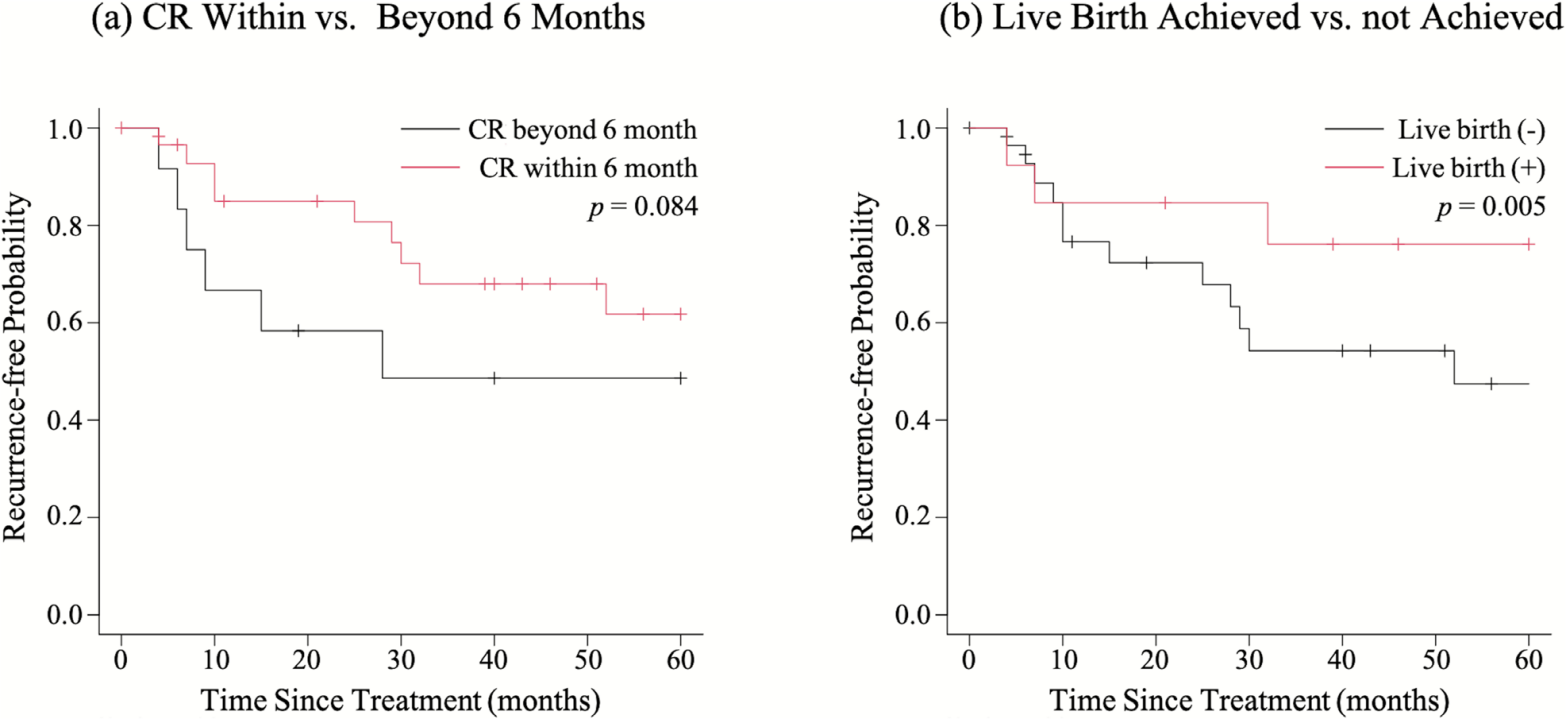
Kaplan–Meier curves of recurrence‐free interval after CR. (a) CR within versus beyond 6 months. (b) Live birth achieved versus not achieved. Patients who achieved CR within 6 months tended to have a longer RFI than those who required more than 6 months. Additionally, patients who achieved live births had a significantly longer RFI than those who did not. CR, complete response; RFI, recurrence‐free interval.

Of the 42 patients who achieved CR after initial treatment, 21 (50%) experienced recurrence. Using the Kaplan–Meier method, RFI was analyzed (Figure 3). Patients who achieved CR beyond 6 months tended to have a shorter RFI than those who achieved CR within 6 months (*p* = 0.08). Moreover, patients who achieved live births had a significantly longer RFI than those who did not (*p* < 0.01).

## 4 Discussion

This study evaluated reproductive outcomes following extended or repeated MPA therapy for fertility‐sparing management of AEH and early low‐grade endometrial carcinoma, incorporating patients' initial desire for prompt conception. One‐third of women who achieved CR within 6 months achieved live births, whereas births after extended or repeated therapy were less common (8%–17%). Although no births occurred after two or more recurrences in our cohort, previous studies have reported favorable outcomes even after multiple MPA courses [11, 12]; therefore, these findings should be interpreted cautiously.

Extended or repeated MPA therapy may remain an option for carefully selected patients when oncologic safety can be ensured. In our cohort, patients for whom hysterectomy was recommended because of inadequate response or pathological progression underwent surgery without recurrence, suggesting that discontinuation of conservative management was appropriately timed. Nevertheless, the balance between preserving fertility and maintaining oncologic control is inherently difficult, as hysterectomy permanently removes reproductive potential, whereas delaying indicated surgery may risk progression.

The two fatal cases further underscore that fertility‐sparing therapy is appropriate only when patients can adhere to close surveillance and accept definitive surgery when medically indicated. In both instances, uterine preservation gradually became the primary goal, diverging from the initial objective of achieving remission and enabling future childbearing. Ensuring that patients fully understand these principles from the outset—and periodically revisiting their reproductive intentions—may support safer and more goal‐aligned treatment trajectories.

Several mechanisms may explain the lower LBR in the extended and retreatment groups. First, these patients underwent more frequent curettage, which may negatively affect endometrial receptivity, although evidence remains mixed [13, 14, 15]. Second, tumors requiring prolonged or repeated treatment may be intrinsically less progestin‐responsive, and molecular alterations contributing to resistance (e.g., PI3K/AKT dysregulation) could also impair receptivity in histologically normal endometrium and are often present even before overt malignant transformation [16, 17, 18]. Third, background differences may have influenced outcomes: the standard group contained more AEH, which typically responds more rapidly and shows longer time to recurrence [19, 20], and women who initially expressed a desire for prompt conception were more likely to pursue infertility treatment. Fourth, fertility preservation requires not only oncologic remission but also reproductive intent, access to treatment, and partner status; therefore, including women without immediate fertility plans—as in our real‐world cohort—naturally lowers overall LBR compared with studies limited to women actively attempting conception [21, 22, 23].

Taken together, these findings suggest that although extending or repeating MPA therapy can occasionally lead to live birth, outcomes are generally less favorable than after standard treatment. Attempting pregnancy early may be advantageous for women ready to conceive [24], yet the timing of CR primarily reflects tumor biology. Thus, counseling should emphasize appropriate patient selection, careful monitoring, and recognition of clinical indicators—such as response kinetics and recurrence patterns—that help determine whether continued conservative therapy is justified.

This study has limitations, including its retrospective single‐center design and small sample size, particularly in the extended and retreatment groups. Owing to the limited statistical power, these subgroup analyses should be regarded as descriptive and hypothesis‐generating rather than confirmatory. The wide confidence intervals and non‐significant *p* values should therefore be interpreted with caution. Molecular classification based on The Cancer Genome Atlas (TCGA) could not be applied because the study period included earlier years [25], and the mismatch repair (MMR) status was unavailable. Given reports of reduced responsiveness to progestin therapy in MMR‐deficient tumors [26], future studies integrating molecular markers are warranted to enable more individualized fertility‐sparing strategies.

In conclusion, extended or repeated MPA therapy can result in live births, although outcomes are generally less favorable than after standard treatment. Careful patient selection, vigilant surveillance, and clear alignment of oncologic and reproductive goals are essential when considering conservative management for young women wishing to preserve fertility.

## Author Contributions

**Taito Miyamoto:** writing – review and editing, methodology. **Mana Taki:** methodology, writing – review and editing. **Koji Yamanoi:** writing – review and editing, methodology. **Akitoshi Yamamura:** investigation, writing – original draft, formal analysis.

## Disclosure

An earlier version of this article was presented at the 69th Annual Meeting of the Japan Society for Reproductive Medicine, which was held in Nagoya, Japan, on 14–15 November 2024.

## Ethics Statement

This study was approved by the Institutional Review Board of Kyoto University (approval number R4535).

## Consent

The authors have nothing to report.

## Conflicts of Interest

The authors declare no conflicts of interest.

## Supporting information


**File S1:** Individual Patient Data. De‐identified individual patient data used for the analyses reported in this article, including baseline characteristics, treatment details, and reproductive outcomes.

**Table S1:** Infertility Treatment Details by Initial Desire for Prompt Conception.

## Data Availability

The data that supports the findings of this study are available in the Supporting Information of this article.
