# Supplementary material for: Live Birth Outcomes After Extended or Repeated High‐Dose Medroxyprogesterone Acetate Therapy for Fertility‐Sparing Management of Endometrial Neoplasia: A Single‐Center Retrospective Case Series
Source: J Obstet Gynaecol Res. 2026 Jan 14;52(1):e70178. doi: 10.1111/jog.70178 (PMC12803870; doi:10.1111/jog.70178)
Supplement: Supplementary file 2 — Table S1: Infertility Treatment Details by Initial Desire for Prompt Conception. [file JOG-52-0-s001.docx]

**Supplementary Table S1. Infertility Treatment Details by Initial Desire for Prompt Conception**

|  | Initial desire  for prompt conception (+)  (*n*=32) | Initial desire  for prompt conception (-)  (*n*=21) |
| --- | --- | --- |
| Infertility treatment after CR: ART  : Non-ART  : None  Not achieved CR | 17 (53)  9 (28)  1 (3)  5 (16) | 3 (14)  2 (10)  10 (48)  6 (29) |

Categorical variables are presented as *n* (%).

CR, complete response; ART, assisted reproductive technology
